# Heme Oxygenase/Carbon Monoxide Participates in the Regulation of *Ganoderma lucidum* Heat-Stress Response, Ganoderic Acid Biosynthesis, and Cell-Wall Integrity

**DOI:** 10.3390/ijms232113147

**Published:** 2022-10-29

**Authors:** Tao Wu, Xiaotian Liu, Ting Wang, Li Tian, Hao Qiu, Feng Ge, Jing Zhu, Liang Shi, Ailiang Jiang, Hanshou Yu, Ang Ren

**Affiliations:** 1 Key Laboratory of Microbiological Engineering of Agricultural Environment, Ministry of Agriculture, Department of Microbiology, College of Life Sciences, Nanjing Agricultural University, Nanjing 210095, China; 2 Sanya Institute of Nanjing Agricultural University, Sanya 572025, China; 3 Institute of Biology, Guizhou Academy of Sciences, Guiyang 550009, China

**Keywords:** heme oxygenase, carbon monoxide, ganoderic acid, heat stress

## Abstract

Carbon monoxide (CO), a product of organic oxidation processes, arises in vivo principally from the enzymatic reaction of heme oxygenase (HO, transcription gene named *HMX1*). HO/CO has been found to exert many salutary effects in multiple biological processes, including the stress response. However, whether HO/CO is involved in the regulation of the heat-stress (HS) response of *Ganoderma lucidum* (*G. lucidum*) is still poorly understood. In this paper, we reported that under heat stress, the *HMX1* transcription level, HO enzyme activity, and CO content increased by 5.2-fold, 6.5-fold and 2-fold, respectively. *HMX1* silenced strains showed a 12% increase in ganoderic acid (GA) content under HS as analyzed by HPLC. Furthermore, according to Western blot analysis of the protein phosphorylation levels, *HMX1* attenuated the increase in phosphorylation levels of slt2, but the phosphorylation levels were prolonged over a 3 h HS time period. The chitin and glucan content in *HMX1* silenced strains increased by 108% and 75%, respectively. In summary, these findings showed that the HO/CO system responds to heat stress and then regulates the HS-induced GA biosynthesis and the cell-wall integrity mediated by the Slt-MAPK phosphorylation level in *G. lucidum*.

## 1. Introduction

Carbon monoxide (CO) is a colorless and odorless gaseous molecule, which has long been considered a toxic gas [1]. In recent years, CO, together with nitric oxide (NO) and hydrogen sulfide (H_2_S), has attracted great attention as the main endogenous gasotransmitter [2,3]. In organisms, CO is mainly produced by heme oxygenase (HO), which is the rate-limiting enzyme in the breakdown of heme into CO, iron, and biliverdin (BV) 4. HO and its product CO play a key role in the redox state of cells by adjusting a variety of stress responses, including oxidative stress [4], drought stress [5], and salinity stress [6,7], etc., thereby maintaining cell and tissue homeostasis. Since the gaseous signaling molecule has become a hot topic, the HO/CO system has attracted more and more attention.

As an inducible isoform in the HO system [8], the expression of HO-1 is highly up-regulated when faced with conditions including but not limited to the above-mentioned environmental stresses in response to possible damage. For instance, HO-1 was involved in the salicylic-acid-induced alleviation of cadmium-triggered oxidative stress by re-establishing redox homeostasis in *Medicago sativa* [9]. Moreover, the HO-1 gene in *Brassica napus* was required for salinity- and osmotic-stress-induced lateral root formation, with a possible interaction with auxin signaling [10]. It was also found that TaMSRA4.1, one of the members of the methionine sulfoxide reductase (MSR) gene family, could interact with HO-1 during the response to salinity and drought stress in wheat [11]. In research on *Paracoccidioides* spp., HSP30 was considered a possible HO recruited to the cell surface to participate in the deprivation of nutrients from the host [12]. Two HO candidates were identified in the fungus *Alternaria alternata*. Despite the poor degree of conservation of HO, it is involved in the biosynthesis of the fungal phytochrome chromophore in mitochondria [13]. These studies suggest that HO plays a regulatory role in responding to the environment and regulating intracellular secondary metabolism, but further research is needed on how HO regulates these physiological processes.

*Ganoderma lucidum*, a well-known medicinal fungus in East and Southeast Asia, contains varieties of active components with pharmacological functions [14,15,16]. Numerous studies have suggested that ganoderic acid (GA) is one of the main active ingredients, which has a wide range of health and therapeutic effects, such as anti-oxidation, anti-inflammatory, anti-tumor, and immune regulation [17,18], etc. How to increase the production of GA, thereby reducing industrial production costs and yielding additional economic benefits, is increasingly attractive. Previous reports have indicated that the accumulation of GA was regulated by many factors, such as heat stress [19], acetic acid [20], methyl jasmonate [21], and sphingolipid [22]. Under heat stress, the growth of mycelia was inhibited, and the GA content was increased [19]. Further research revealed reactive oxygen species (ROS) bursts under heat stress, which were the reason for the increase in GA content [23]. Whether the HO/CO system can respond to heat stress and plays an important role in *G. lucidum* remains unclear.

We previously explored the enzymatic activity of HO and cloned the HO gene *HMX1* in *G. lucidum*. Silencing strains of *HMX1* were also constructed and named *HMX1i1* strain and *HMX1i2* strain. The previous study also found that HO had certain effects on the growth of mycelia and polysaccharide synthesis in *G. lucidum* [24]. In this study, silencing of *HMX1* under heat stress was found to significantly increase the GA content, and exogenous addition of activators and inhibitors of HO also affected the GA biosynthesis. In addition, silencing of *HMX1* was found to affect the cell-wall sensitivity and content of chitin and glucan in this study. By investigating the response mechanism of the HO/CO system under heat stress, we further provide a theoretical basis for improving the polysaccharide and triterpene contents of *G. lucidum* and provide a reference for other species to study the function of *HMX1*.

## 2. Results

### 2.1. HMX1 Transcription Level, HO Enzyme Activity, and CO Content Were Increased under Heat Shock

In this study, several stresses that were previously reported to be able to affect ganoderic acid in *G. lucidum* were chosen to detect changes in the transcript levels of *HMX1*. These include physical factors such as heat stress [19]; the chemical additives acetic acid [20], ethylene [25], aspirin [26], methyl jasmonate [21], and salicylic acid [27]; and different types and concentrations of nitrogen source culture [28]. The transcript level of *HMX1* was highly significantly increased by 6.2-fold under heat stress for 30 min in supplementary material (Figure S1A). Treatment with acetic acid, ethylene, aspirin, methyl jasmonate, and salicylic acid failed to significantly increase the transcript levels of *HMX1* (Figure S1B,C). The transcript levels of *HMX1* of WT strains incubated with 30 mM ammonium salt with 30 mM glutamine as the only nitrogen source did not change significantly, but the transcript levels of *HMX1* incubated with low concentrations of ammonium salt all increased 2.2-fold. Taken together, these results revealed that the *HMX1* transcript levels changed most significantly under 30 min of heat-stress conditions; hence, we speculate that HO may be involved in the process of heat stress affecting GA biosynthesis. Therefore, heat stress that can significantly affect GA biosynthesis was used as the stress condition in the next study.

**Figure 1:**
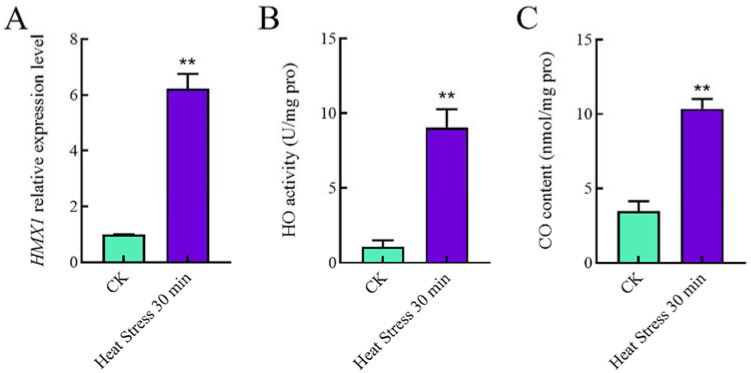
Changes in *HMX1* transcription level, enzyme activity, and CO content under heat shock. (**A**) The relative expression level = transcription level under heat shock treatment/transcription level under control condition (without heat shock). (**B**) HO activity was significantly increased after 30 min of heat shock. (**C**) CO content was significantly increased under heat shock for 30 min. Error bars are represented as mean ± SEM. Significance was accepted at ** *p* < 0.01. The same as follows.

To determine changes in HO/CO under heat stress, the *HMX1* transcript levels, HO activity, and CO content were analyzed. Under heat shock, the *HMX1* transcript levels were significantly increased by 5.2-fold (Figure 1A). HO activity after heat shock was 6.5-fold higher than without heat shock (Figure 1B). Furthermore, the CO level after heat shock was 2.0-fold higher than without heat shock (Figure 1C). Under heat stress, the *HMX1* transcript level, enzymatic activity, and CO level increased, indicating that *HMX1* has a specific response to heat stress.

### 2.2. Silencing of HMX1 Reduces the Ability of Mycelium to Restore Growth under Heat Shock

**Figure 2:**
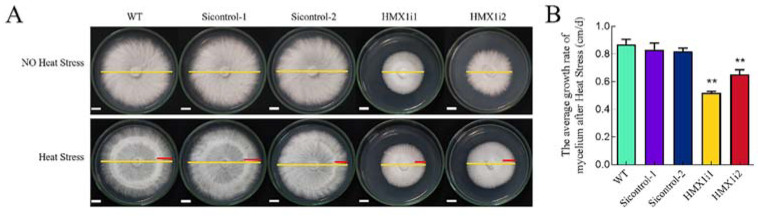
Comparison of mycelial growth under heat stress. (**A**) Under the circumstance of non-heat stress or heat stress, the growth of WT, Si-control, and *HMX1* silenced strains in 5 days. The yellow line is the diameter of the mycelium after 7 days of growth on plate; after 5 days of mycelium growth on plate, the red line is the distance that the mycelium recovered for 2 days after heat shock at 42 °C for 30 min. The white line is the scale bar, scale bar = 1 cm. (**B**) The average mycelium growth rates of WT, Si-control, and *HMXli* strains 2 days after heat shock, respectively. Error bars are represented as mean ± SEM. Significance was accepted at ** *p* < 0.01. The same as follows.

According to previous studies [19], the growth rate of *G. lucidum* mycelium slowed down under heat stress. Therefore, the ability to restore mycelium growth after heat shock was further investigated in *G. lucidum* WT, Si-control, and *HMX1* silenced strains. The results showed that the average growth rate of the hyphae of the WT strain after heat-stress treatment was 0.9 cm/d, and the average growth rates of the hyphae of the *HMX1i1* and *HMX1i2* strains were 0.5 cm/d and 0.65 cm/d, respectively (Figure 2A,B). The average growth rate of the *HMX1* silenced strain was significantly lower than WT, while Si-control strains had no significant differences compared with WT. These findings showed that silencing *HMX1* weakened the ability of mycelium to restore growth after heat stress.

### 2.3. HO Affected GA Content under Heat Shock

**Figure 3:**
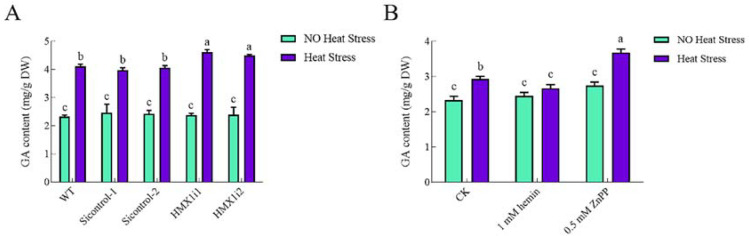
Effects of HO on GA content under heat stress. (**A**) GA content of *HMX1* silenced strains was significantly higher than that of WT strain under heat stress. (**B**) Exogenously adding 1 mM hemin did not affect GA content; in other words, the increasing trend of GA content under heat stress compared with non-heat stress was suppressed. In contrast, the content of GA was significantly increased in WT strain treated with 0.5 mM exogenous ZnPP under heat stress. All calculated results are expressed as mean ± standard deviation, and these English letters of “a”, “b” and “c” are indicated significant differences among different treatments (*p* < 0.05).

Our previous work reported that the GA content was significantly increased under heat stress [19,29]. The results showed that there was no significant difference in GA content between *HMX1* silenced strains and WT or Si-control strains without heat-shock treatment. While under heat stress, the GA content of *HMX1* silenced strains was significantly higher than that of the WT strain, with a value of 12% (Figure 3A).

To further verify the relationship between HO and GA biosynthesis, the addition of hemin, an activator of HO, to the WT strain was used to increase HO activity, while the inhibitor zinc protoporphyrin IX (ZnPP) was used to inhibit HO activity. Under non-heat stress, there was no significant change in GA content regardless of the addition of hemin or ZnPP. Under heat stress, the GA content was significantly increased by 26% with the addition of Znpp compared to the control group, but the exogenous addition of 1 mM hemin inhibited this increasing trend (Figure 3B). On the contrary, the GA content increased by 34% after 0.5 mM exogenous ZnPP treatment of the WT strain (Figure 3B). The above results indicated that HO could play a negative regulatory role in the process of accumulation of GA under heat stress.

### 2.4. CO and Bilirubin Affected the Biosynthesis of GA under Heat Shock

**Figure 4:**
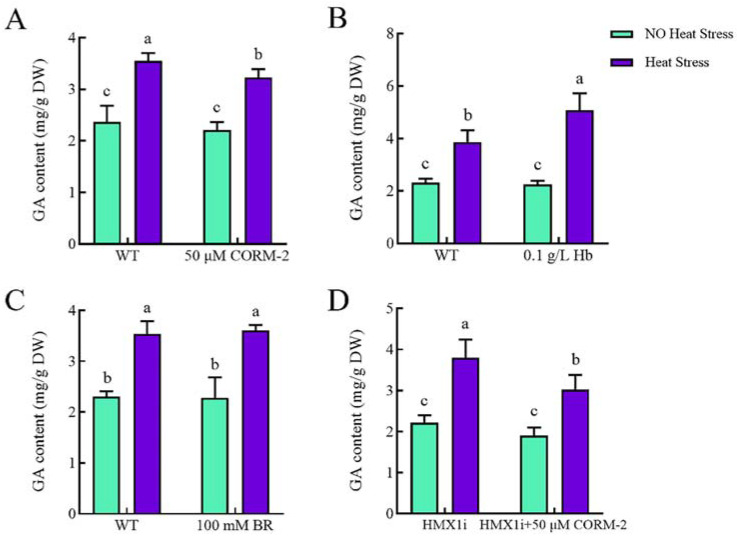
Effects of CO and BR on GA content under heat stress. Under heat stress, GA content would be significantly reduced or increased compared with the addition of the same dose of DMSO provided (**A**) 50 μM CORM-2 or (**B**) 0.1 g/L Hb was added into WT strain, respectively. (**C**) The addition of 100 mM BR to WT strain would not affect GA content. (**D**) The addition of 50 μM CORM-2 to *HMX1* silenced strains diminished the GA content under heat stress. All calculated results are expressed as mean ± standard deviation, and these English letters of “a”, “b” and “c” are indicated significant differences among different treatments (*p* < 0.05).

HO is a rate-limiting enzyme which degrades heme into Biliverdin (BV), CO, and iron. BV is then readily reduced to bilirubin (BR) due to BV reductase. Besides HO, the products CO and BR can also play important roles in some biological processes [4,30]. Therefore, we speculated that CO and BR could be involved in the regulation of GA biosynthesis in *G. lucidum*. Different concentrations of haemoglobin (Hb), a scavenger of CO, were added to WT strains to exclude the effect of CO on GA biosynthesis under non-heat stress and heat stress; CO-releasing molecules-2 (CORM-2), a donor of CO, was also added to WT strain and *HMX1* silenced strains to verify the effect of CO on GA biosynthesis, as well as to adding BR and verifying the effect of BR on GA biosynthesis. Whether adding 50 μM CORM-2 or 0.1 g/L Hb to the WT strain, the GA content would not be significantly altered under non-heat stress. The GA content of WT strains treated with CORM-2 (Figure 4A) or Hb (Figure 4B), respectively, was only significantly reduced by 9% or increased by 31% under heat shock compared to the addition of the same dose of dimethylsulfoxide (DMSO). However, whether under heat stress or non-heat stress, the addition of 100 mM BR would not affect the GA content (Figure 4C). Similarly, the addition of 100 mM BR also did not affect the GA content in *HMX1i* strain under heat stress or non-heat stress (Figure S2). Moreover, the addition of 50 μM CORM-2 to *HMX1* silenced strains further revealed that the GA content was decreased by 20% under heat stress (Figure 4D). These findings indicate that under heat stress, CO negatively regulated GA biosynthesis, while BR was not involved in this process.

### 2.5. Silencing of HMX1 Affected the slt2 MAPK and Cell Wall

**Figure 5:**
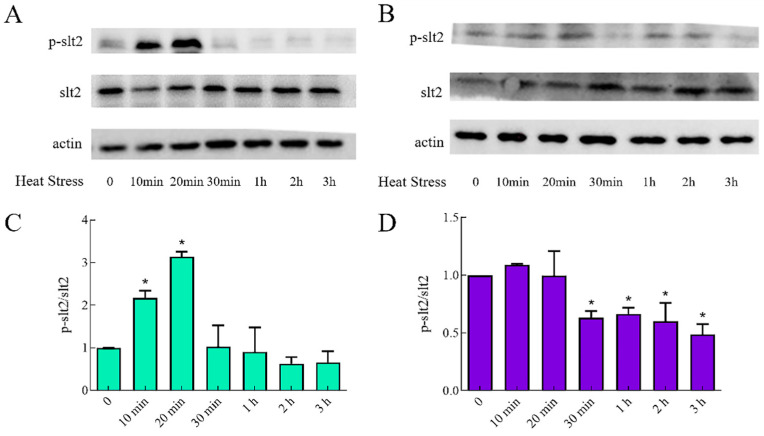
Effects of Silencing *HMX1* on slt2 MAPK. The p-slt2/slt2 of WT and *HMX1i* strains under different heat shock times were as illustrated in (**A**,**B**), respectively. Quantification of p-slt2/slt2 levels in WT and *HMX1i* strains at different heat shock times are shown in (**C**,**D**). Error bars are represented as mean ± SEM. Significance was accepted at * *p* < 0.05. The same as follows.

It was reported that CO exerted its biological function via several pathways including MAPK [31,32]. Given that slt2 was part of the MAPK pathway [33] and its activation status was traditionally monitored by calculating the ratio of phosphorylated protein to corresponding total (phosphorylated and unphosphorylated) protein, we investigated the p-slt2/slt2 of WT and *HMX1i* strains under different heat-shock times, respectively. The results show that during heat stress, p-slt2/slt2 in WT increases until it peaks at 20 min and then decreases, which indicated that the slt2 of *G. lucidum* could respond to heat stress (Figure 5A). In contrast, p-slt2/slt2 in the *HMX1* silenced strain showed a decreasing trend in response to heat stress, reaching a minimum level at 3 h. The degree of change in p-slt2/slt2 was reduced in the *HMX1*-silenced strain compared to WT, but the response time was longer (Figure 5B). The above results showed that silencing *HMX1* would affect the phosphorylation status of slt2 under heat stress, implying that *HMX1* could respond to heat stress by altering the phosphorylation status of slt2.

**Figure 6:**
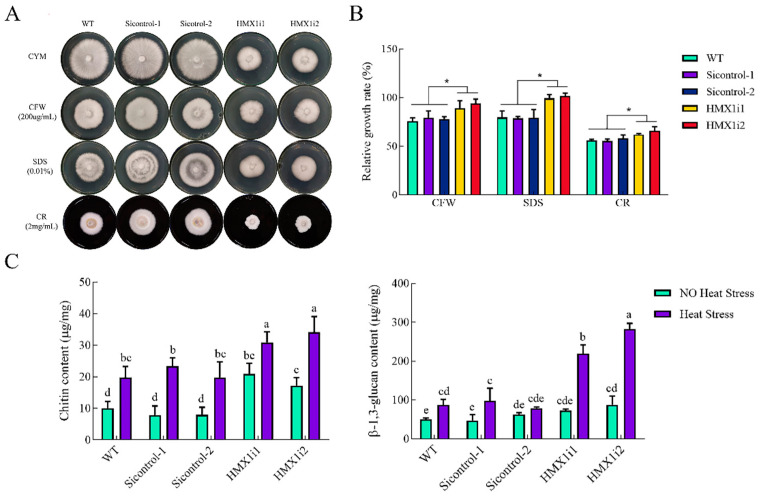
Effects of Silencing *HMX1* on cell wall susceptibility and components. (**A**) WT, Sicontrol, and *HMX1* silenced strains cultured on medium supplied with 200 μg/mL CFW, 0.01% SDS, or 2 mg/mL CR, respectively, (**B**) and their corresponding relative growth rate. (**C**) The chitin and glucan content in the mycelia of WT, Sicontrol, and *HMX1* silenced strains under heat stress or non-heat stress, respectively. All calculated results are expressed as mean ± standard deviation, and these English letters of “a”, “b”, “c”, “d” and “e” are indicated significant differences among different treatments (*p* < 0.05). Significance was accepted at * *p* < 0.05.

Previous studies have demonstrated that slt2 plays a key role in the cell-wall integrity (CWI) pathway [15]. Since calcofluor white M2R (CFW), sodium dodecyl sulfate (SDS), and congo red (CR) are commonly used to detect cell-wall stress sensitivity, these reagents were used in this study to investigate the effect of *HMX1* silencing on CWI (Figure 6A). The relative growth rate of WT under the addition of CFW was 76% compared to the control group, while the relative growth rates of *HMX1i1* and *HMX1i2* strains were 89% and 94%, respectively (Figure 6B). The relative growth rate of WT in SDS-supplemented medium was 80%, and the relative growth rates of *HMX1i1* and *HMX1i2* strains were 99% and 100%, respectively (Figure 6B). When CR was added to the medium, the relative growth rates of *HMX1i1* and *HMX1i2* strains were 62% and 66%, respectively, which were significantly higher than WT (Figure 6B). These data together suggested that silencing *HMX1* reduces sensitivity to cell-wall stress under treatment with the above-mentioned interfering substances.

The reason for this weakened susceptibility of *HMX1* silenced strains to cell-wall stress may be the content of cell-wall components, which consist mainly of chitin and glucan [15,34]. Therefore, the mycelial content of chitin and glucan in the mycelium of WT, Sicontrol and *HMX1* silenced strains was measured separately. After 7 days of growth, the chitin contents of the *HMX1i1* and *HMX1i2* strains were increased by 108% and 71% compared with WT, respectively. Moreover, the glucan content of the *HMX1i1* and *HMX1i2* strains were increased by 45% and 75% compared with WT, respectively (Figure 6C). The above results indicated that silencing *HMX1* could increase the content of *G. lucidum* cell-wall components.

Furthermore, after heat shock at 42 °C for 30 min, the chitin and glucan content of the WT strain were significantly increased by 97% and 73%, respectively. Compared with WT, the chitin contents of the *HMX1i1* and *HMX1i2* strains were increased by 56% and 72%, respectively, and the glucan contents of the *HMX1i1* and *HMX1i2* strains were increased by 152% and 225%, respectively (Figure 6C). These results indicated that the content of chitin and glucan would be significantly increased after heat shock, and silencing *HMX1* would further enhance this process, which implied that *HMX1* in *G. lucidum* could be involved in the process of slt2 affecting cell-wall components and responding to heat stress.

## 3. Discussion

Organisms have evolved a variety of strategies to respond to diverse environmental stresses. CO has become an important signaling mediator in a variety of biological processes, including the modulation of multiple stress responses. Endogenous CO arises principally from the enzymatic reaction of HO [35], which catalyzes the rate-limiting step in heme degradation [36]. HO/CO has been reported to be involved in responses such as drought stress to promote developmental processes and maintain homeostasis in plants [37], including anti-injury [38] and antioxidant defense effects [39] in animals. As one of the environmental stresses, heat stress has been used to study the function of HO/CO in the modulation of resistance and secondary metabolism. As expected, the transcript levels of *HMX1*, enzymatic activity of HO and the content of CO increased fivefold, sixfold and twofold, respectively, after heat, which indicated that HO in *G. lucidum* could also be induced by stress. Which means, the results of the heme oxygenase vitro enzyme activity assay in *G. lucidum* showed that HO plays a similar enzymatic function to that of HO in animals [24]. 

GA biosynthesis is a complex physiological and metabolic process, containing huge regulatory networks. Some of the mechanisms by which external and internal factors promote the accumulation of GA under heat stress have been interpreted in previous work [19,29,40,41]. This study showed that HO can also respond to heat stress, and its silencing contributed to a 12% increase in GA content, despite a diminished ability of the mycelium to resume growth. Recently, it has been reported that HO could interact with phytochrome and was involved in metabolic pathway of chromophore assembly at mitochondria in *Alternaria alternate* [13]. The mycelium of *G. lucidum* was treated with hemin, carbon monoxide-releasing molecule-2 (CORM-2) and Zn protoporphyrin IX (ZnPPIX), a specific HO-1 inhibitor. The results showed that the GA content increased by 60.1% and 56.0% and decreased by 26.0%, while the enzymatic activity of HO increased by 57.1% and 18.1% and decreased by 15.8%, respectively [42]. In addition to fungi, HO was vital for modulating glucose and energy metabolism in mammals [43]. In this study, we speculated that HO could participate in the regulation of GA content through its catalytic product CO.

Many studies have pointed out the association between CO and MAPK [31,44]. In the present study, we also found that the phosphorylation level of slt2 MAPK gradually decreased to the lowest level within 3 h under heat stress in *HMX1* silenced strains. Given the fact that CO could negatively regulate GA biosynthesis and silencing slt2 could affect GA biosynthesis [34], we can reasonably speculate that CO responds to heat stress through slt2 MAPK, resulting in this negative regulation of GA content. Moreover, since slt2 was part of the CWI pathway, the effect of silencing *HMX1* on the cell-wall composition and susceptibility to cell-wall stress was examined. The chitin and glucan contents increased by 97% and 73%, respectively, which may explain the reduced sensitivity of *HMX1* silenced strains to cell-wall stress. A recent study showed that HSP30, which was a possible HO in the *Paracoccidioides* genus and could be recruited on the cell surface, was important for *P. brasiliensis* to use host hemoglobin as an iron source [12]. These studies all suggest that HO/CO has an important role in regulating cell-wall components and functions. To sum up, we revealed that HO/CO could respond to heat stress by altering the phosphorylation status of slt2, maximizing the chitin and glucan content by 72% and 225%, respectively, while participating in the regulation of the *G. lucidum* heat-stress response, GA biosynthesis, and cell-wall integrity. 

## 4. Materials and Methods

### 4.1. Strains and Culture Conditions

The dikaryotic *G. lucidum* strain ACCC53264 (obtained from the Agricultural Culture Collection of China) was used as the wild-type (WT) strain. *HMX1* silenced strains were constructed previously [24]. Under normal conditions, these *G. lucidum* strains were cultured in CYM medium (10 g/L maltose, 20 g/L glucose, 4.6 g/L KH_2_PO_4_, 2 g/L tryptone, 2 g/L yeast extract, and 0.5 g/L MgSO_4_·7H_2_O) at 28 °C and 150 rpm.

### 4.2. Treatment of Mycelium

According to a previous study [19], 42 °C was adopted as the temperature of heat-stress treatment. In terms of heat-shock treatment, the corresponding *G. lucidum* strains were cultured in the CYM liquid medium at 28 °C and 150 rpm for 5 days, subsequently exposed to 42 °C for 12 h, and then statically cultured at 28 °C for another 36 h. If necessary, 1 mM hemin, 0.5 mM zinc protoporphyrin IX (ZnPP), 50 μM CO-releasing molecules-2 (CORM-2), or 0.1 g/L hemoglobin (Hb) was added to the fermentation broth under aseptic conditions after shaking culture at 28 °C for 5 days, followed by shaking culture at 28 °C for 30 min, and followed by being exposed to 42 °C for 12 h. The control groups (CK) were administered the same dose of dimethyl sulfoxide (DMSO). Experiments were repeated three times independently with three replicates per experiment.

### 4.3. Quantitative Real-Time PCR (qRT-PCR)

The total RNA of *G. lucidum* was extracted (RNAiso^™^ Plus Reagent, TaKaRa, Dalian, China) and reverse transcribed into cDNA (PrimeScript^®^ RT reagent Kit, TaKaRa) according to the manufacturer’s instructions. Quantitative real-time PCR (qRT-PCR) was performed with SYBR green fluorescence and monitored by Eppendorf Mastercycler ep realplex 2.2 software. The expression level of *HMX1* was normalized to the amount of the housekeeping gene 18S rRNA (18S). The 2^−ΔΔCT^ method described by Livak and Schmittgen [45] was adopted to analyze the relative quantification of gene expression. Three independent biological replicates and three technical replicates of each sample were made for quantitative PCR analysis. The specific primers used in this experiment were listed in Table S1.

### 4.4. Detection of Enzyme Activity

The detection of HO activity in *G. lucidum* was performed as described previously [46] with minor modifications. Briefly, *G. lucidum* mycelia (0.2 g) were ground in liquid nitrogen and then added to 30 mL of pre-cooled isolation medium containing 25 mM HEPES-Tris (pH 7.4), 250 mM mannitol, 1% (*w*/*v*) polyvinylpyrrolidone, 1 mM EDTA, 10% (*v*/*v*) glycerol, and 1 mM dithiothreitol. The crude enzyme extract was obtained after being filtered by non-woven fabric and centrifuged at 12,000× *g* for 30 min at 4 °C. For HO activity determination, a total volume of 80 μL consisting of 20 μL of enzyme extract, 10 μM hemin, 0.15 mg/mL bovine serum albumin, 50 μg/mL spinach ferredoxin, 5 mM ascorbate, 0.025 units/mL spinach ferredoxin-NADP^+^ reductase, and 2 mM desferrioxamine in 100 mM HEPES-NaOH (pH 7.2) was prepared. Adding NADPH to a final concentration of 100 μM represented the starting point of the reaction, and then the samples were incubated at 37 °C for 30 min. The concentration of BV was evaluated via a molar absorption coefficient at 650 nm of 6.25 mM^−1^ cm^−1^ in 0.1 M HEPES-NaOH buffer (pH 7.2). One unit of activity (U) was defined as the quantity of the enzyme to produce 1 nM BV per 30 min.

### 4.5. Detection of CO Content

The method for determining the CO content was performed as described previously [47] with minor modifications. In short, approximately 0.2 g of mycelia were ground in liquid nitrogen and added to 1 mL of H_2_O. After centrifuging at 3000× *g* for 5 min at room temperature, supernatants (0.5 mL) of samples were mixed with 0.5 mL of 1 mg/mL hemoglobin (Hb), which was dissolved in 0.24 M ammonia solution, and then 0.1 mL of 0.2 g/mL freshly prepared sodium dithionite solution was added. After 10 min of reaction, the ratio of the absorbance at 420 and 432 nm was calculated to estimate the % HbCO [48].

### 4.6. Detection of GA Content (HPLC)

GA was extracted as described before [49], and high-performance liquid chromatography (HPLC) was used for the quantification of GA. The Prominence-i LC-2030 plus apparatus from Shimadu Corporation, which was equipped with a Shim-pack VP-ODS C18 column (4.6 mm × 250 mm, 5 μm), was used. The mobile phase A was 100% acetonitrile and mobile phase B contained ultra-pure water/acetic acid (10,000:1 *v*/*v*). A 20 min linear gradient from 50% A to 100% A at a constant flow rate of 1 mL/min was adopted. The procedure was monitored at a wavelength of 252 nm.

### 4.7. Determination of Mycelial Growth Rate and Cell Wall Stress Treatment

The diameters of corresponding mycelia were measured before and after heat-shock treatment, respectively, and their growth rates were also calculated.

*G. lucidum* strains cultured on CYM solid medium were supplied with, if needed, 200 μg/mL calcofluor white M2R (CFW), 0.01% sodium dodecyl sulfate (SDS), or 2 mg/mL congo red (CR), respectively. The growth status on different medium was observed and measured.

### 4.8. Detection of β-1,3-D-Glucan and Chitin in the Cell Wall

The detection of β-1,3-D-glucan content was carried out according to the previous aniline blue assay [50], while a chitin assay was performed based on the previous protocol [34,51].

### 4.9. Western Blot

The slt2 phosphorylation status in *G. lucidum* was analyzed by Western blot using antibody against the dually phosphorylated forms of p44/42 MAPK (mitogen-activated protein kinases) according to the methods described in our previous study [34].

### 4.10. Statistical Analysis

Data from at least three independent measurements were averaged. Error bars were represented as mean ± SEM. The significance of differences was determined by one-way analysis of variance ((ANOVA) for three or more samples) or Student’s *t*-test (for two samples). Significance was accepted at * *p* < 0.05 or ** *p* < 0.01. Letters were used to indicate statistical significance (*p* < 0.05), and a shared letter meant no significant difference.

## 5. Conclusions

In the present study, HO in *G. lucidum* can be thermally induced by stress; however, how HO responds to heat stress, in other words, what the regulatory molecules located upstream of HO in *G. lucidum* are, is the gap we need to fill. In addition, HO may be involved in the regulation of GA content through its catalytic product CO. Whether HO can directly interact with regulatory elements or enzymes involved in the GA biosynthetic pathway and the mechanisms of modification or regulation of downstream targets by CO remain largely unknown. Most importantly, this study is an expansion of the physiological and metabolic studies of *G. lucidum* under heat stress, and further studies to systematically understand these processes are extremely important to follow.

## Data Availability

Data is contained within the article or supplementary material.

## Supplementary Materials

The following supporting information can be downloaded at: https://www.mdpi.com/article/10.3390/ijms232113147/s1.
