# Supplementary material for: Heme Oxygenase/Carbon Monoxide Participates in the Regulation of Ganoderma lucidum Heat-Stress Response, Ganoderic Acid Biosynthesis, and Cell-Wall Integrity
Source: Int J Mol Sci. 2022 Oct 29;23(21):13147. doi: 10.3390/ijms232113147 (PMC9659044; doi:10.3390/ijms232113147)
Supplement: Supplementary file 1 [file ijms-23-13147-s001.zip › ijms-1981898-supplementary.pdf]

Table S1. Primers used in this study.

Primers	Sequence (5'-3')	Description
RT-HMX1-F	CGTCCCCTCCGTCTACT	Detect *HMX1* expression
RT-HMX1-R	CGGTGCTATTTGCGTTA	
RT-18S-F	TATCGAGTTCTGACTGGGTTGT	Detect 18S expression
RT-18S-R	ATCCGTTGCTGAAAGTTGTAT	

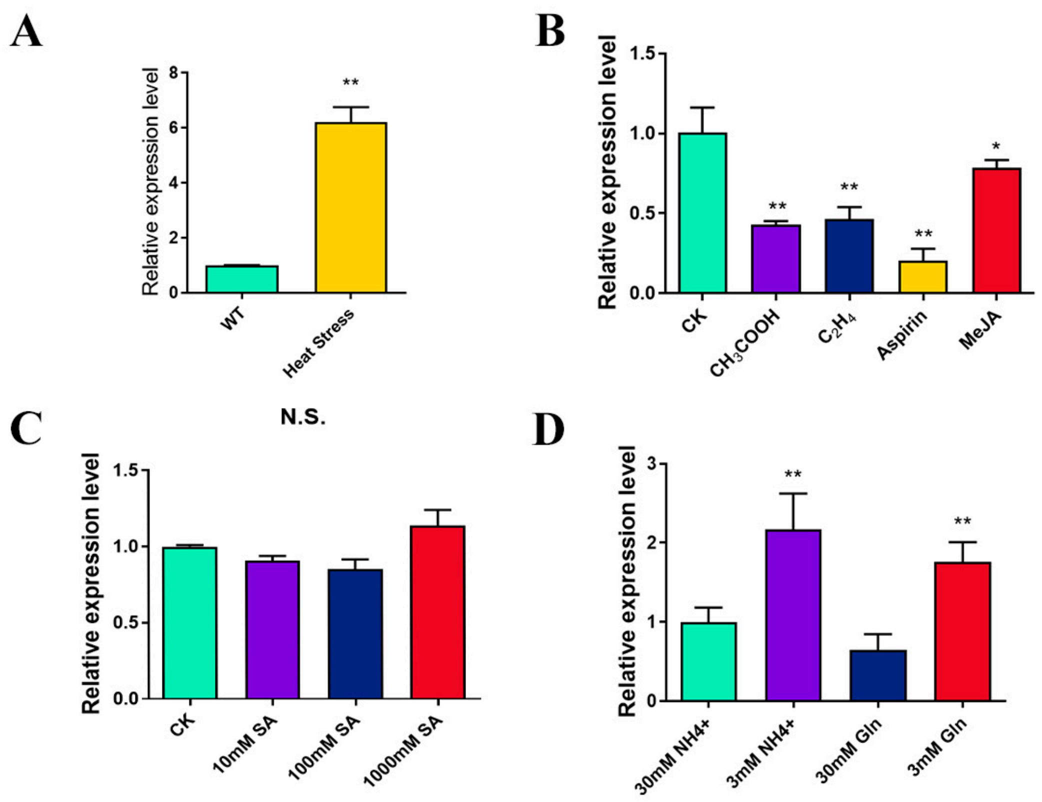

Figure S1. Effect of different treatments on *HMX1* strain transcription level.

(A) The effect of heat shock for 30 minutes on the transcription level of *HMX1*; (B) The effects of exogenous chemical additives acetic acid, ethylene, aspirin and methyl jasmonate on the transcription level of *HMX1*; (C) The effect of exogenous salicylic acid on the transcription level of *HMX1*; CK is the solvent ethanol with the same dose of salicylic acid as the control; (D) The effects of different species and concentration of nitrogen sources on the transcription level of *HMX1*. Error bars are represented as mean ± SEM. Significance was accepted at *P <0.05 or **P <0.01; N.S. is Not Significant.

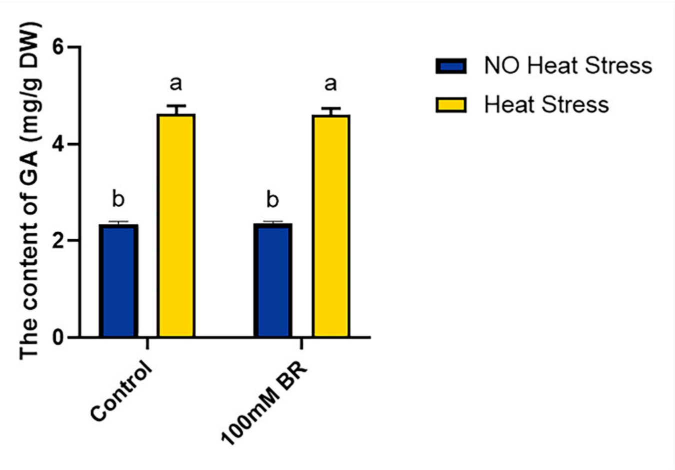

Figure S2. Effects of BR on GA content under heat stress.

The addition of 100 mM BR to *HMX1* strain would not affect GA content. All calculated results are expressed as mean ± standard deviation, and these English letters of “a” and “b” are indicated significant differences among different treatments ($p < 0.05$).
